# Therapeutic stance towards persons with psychosis – a Grounded Theory study

**DOI:** 10.1080/17482631.2024.2333064

**Published:** 2024-03-29

**Authors:** Laura Galbusera, Ralph Endres, Thelke Scholz, Emilia Jirku, Samuel Thoma

**Affiliations:** a Department of Psychiatry and Psychotherapy, Immanuel Klinik Rüdersdorf, Brandenburg Medical School, Rüdersdorf, Germany; b Independent Researcher, Germany; c Department for Social Psychiatry, University Medicine Halle (Saale), Halle, Germany

**Keywords:** Psychotherapy of psychosis, therapeutic stance, ambivalence, openness, dialogical process, Grounded Theory

## Abstract

**Objective:**

Over the last decades, psychotherapy of psychosis has increasingly gained attention. The quality of the therapeutic alliance has been shown to have an impact on therapy outcome. Yet, little is know about the influence of the therapeutic stance on the alliance. In this study, we explore psychotherapists‘ stance towards persons with psychosis with the aim of better understanding its characteristic—hindering and helpful—aspects.

**Method:**

6 semi-structured interviews with psychotherapists from three different schools (CBT, PD, ST) were analysed with Grounded Theory. Credibility was checked through external and peer-researcher-supported debriefing.

**Results:**

4 core categories were generated and interrelated in a theoretical model. Therapists‘ stance was initially characterized by insecurity. Diffent ways of dealing with insecurity yielded different stances: a monological and an open one. A helpful stance was conceived as stemming from openness and was characterized by a dialogical structure. A co-presence (or „dosing“) of you and I was conceived as its core aspect.

**Conclusion:**

These findings specify the interpersonal dynamics arising from different stances and their impact on the therapeutic alliance and process. Research is still needed to further understand the characteristics of helpful and hindering therapeutic stances, which should also inform the training of psychotherapists.

## Introduction

Until the end of the 20^th^ century, the treatment of psychosis consisted predominantly of psychopharmacology. Such a biological focus might be viewed as conceptually rooted in Jaspers (1997) claim of the incomprehensibility of schizophrenic experience. Although Jaspers believed that the (e.g., persecutory) content of delusions could be traced to the patients’ biography, he firmly asserted that the actual emergence of delusions—i.e., the fundamental structural alteration of experience—could not be psychologically understood but only be neurobiologically explained. Ever since, there was a certain tendency to consider talking with psychotic patients about their delusions as medical malpractice (Kulenkampff, 1997). The neurobiological position gained widespread support during the so-called “decade of the brain” that aimed to explore physiological correlates and causes for mental disorders. However, the findings of such research endeavours remain largely heterogeneous until today and their translation into effective treatment methods for clinical practice is still unsatisfying.

Treatment of psychosis has remained mainly pharmaceutical while psychotherapy has not been included in the clinical guidelines and recommendations for psychosis for a long time. Only in the last decades has psychotherapy for psychosis been recognized for its clinical efficacy (Pfammatter et al., 2006) and included in the gold standards for psychiatric care (e.g., inclusion in 2014 in the NICE Guidelines and in 2016 in the German national health insurance system). This growing interest in psychotherapy for psychosis led to the development of specific therapeutic methods and clinical training for psychotherapy of psychosis. In Germany, these new treatment methods and training programmes were developed especially in the three main psychotherapy approaches that are covered by health insurance, i.e., psychodynamic (PD), cognitive-behavioural (CBT) and systemic psychotherapy (ST). Despite an increased research focus on this topic over the last years, research on the effectiveness of psychotherapy for psychosis remains limited. Given this vivid evolution, we are thus currently in need of a better and more comprehensive understanding of the working mechanisms and outcomes of psychotherapeutic interventions for psychosis.

One central aspect that has emerged in the literature on psychotherapy for psychosis so far, is the positive link between the quality of the therapeutic alliance and treatment outcomes (Frank & Gunderson, 1990; Goldsmith et al., 2015; Shattock et al., 2018). From a more general point of view, supportive social relations have been shown to play an essential role in recovery from psychosis (Davidson, 2003). One might therefore conclude that, especially in the case of psychosis, supportive and positive relations rather than specific therapeutic interventions have proven particularly effective (cf. Bourke et al., 2021). Yet, one may ask what specific qualities and aspects characterize a beneficial therapeutic alliance in the context of psychotherapy of psychosis.

The therapeutic alliance as a common helpful factor has been widely investigated in the field of psychotherapy research, beyond the specific issue of psychosis treatment. The relation between the quality of the therapeutic alliance and therapy outcome has been empirically demonstrated in several studies (Orlinsky et al., 2004).

Hewitt and Coffey (2005) summarized that patients experiencing the therapeutic relationship as being helpful tend to show more favourable outcomes. Irrespective of the psychotherapeutic approach, different therapist- and therapy-related factors have been reported to be associated with a better therapeutic relationship. Those factors include genuineness, flexibility, availability, as well as empathy (da Costa et al., 2020; Shattock et al., 2018). Moreover, a higher patient-perceived competence, attractiveness and trustworthiness of the therapist might foster a better working alliance, at least with regard to CBT (Evans-Jones et al., 2009; Jung et al., 2015). Therapists’ confidence in their skills or clinical experience was not related to a better therapeutic relationship (Evans-Jones et al., 2009; Mulligan et al., 2014). In contrast, a relationship that is marked by mostly operational, indifferent, paternalistic and pessimistic characteristics is generally rejected by patients (Ljungberg et al., 2016). Farrelly and Lester (2014) identified three core elements in their integrative model of beneficial therapeutic relationships: mutual trust and respect as well as shared decision-making. A particularly interesting notion is the conflicting role of the therapists who are both obliged to the patient as stakeholders but also committed to the mental health care structures, guidelines and subjected to the general laws (Ljungberg et al., 2016).

Studies have also shown that other personal aspects such as the therapist’s attachment style (Black et al., 2005), her personality traits (Heinonen et al., 2014), her interpersonal history and her self-concept (Lingiardi et al., 2018) have an important impact on treatment outcome of psychotherapy in general.

These findings point on the one hand to the therapist’s specific skills and competencies, which seem to contribute to a working alliance. On the other hand, a therapist’s more personal characteristics such as personality and interactional style also seem to play a crucial role in the construction of the therapeutic relationship but cannot be understood as mere skills. Thus, a broader conceptual frame must be drawn that includes but also goes beyond specific therapeutic competencies and that considers the more encompassing expression of the therapist’s person and its influence on the therapeutic alliance. To better grasp the effect of a therapist’s contribution as a person (in its different facets and complexity) to the construction of a positive therapeutic relationship for psychotherapy of psychosis, some authors have suggested to focus on the concept of *therapeutic stance* (Galbusera et al., 2021). Indeed, through her participation as a person in the clinical situation, the therapist always assumes a particular *stance*, which includes e.g., competencies, beliefs, emotions, etc. (ibid.).

The therapeutic stance is a commonly referred to concept in psychotherapy research. However, its use seems often vaguely described (see e.g., Lingiardi, 2013; Lundh, 2012). We thus hereby refer to Kurbacher (2017) definition of *stance* (Ger.: *Haltung*) as a person’s way of relating to the world and to others, including rational and ethical attitudes and more fundamentally emotional, perceptual and cognitive dispositions. This concept provides us with a sufficiently large—and yet defined—scope for our investigation, i.e., to focus on both cognitive and emotional, conscious and unconscious, active and passive dispositions in the therapeutic relationship. Accordingly, we conceive the therapeutic stance as something more fundamental than therapeutic skills and techniques. The latter two can be learned from handbooks and clinical training and then be selected and performed deliberately. The therapeutic stance—based on Kurbacher’s philosophical perspective—is something that more broadly includes cognitive, emotional and even bodily dispositions, which cannot unilaterally be steered or selectively applied by the person. It is at the same time always inter-personal, in that it is influenced by and directly evokes specific actions and reactions of the other person (cf. Galbusera et al., 2021). Kurbacher (2017) emphasized that a *personal* stance inevitably expresses us as persons. We thus assume that, even if the *therapeutic* stance is related to a specific professional context, it still bears a holistic expression of the therapist’s person, beyond her mere professional identity.

Especially because as a person, the therapist is conveyed through her stance, we believe that the stance might play a particularly important role in the treatment of psychosis. According to some phenomenological authors, the personhood—and more specifically, the self—is especially challenged during psychotic experiences (see e.g., Fuchs, 2000). Indeed psychosis questions the everyday and immediate sense of being-a-person, of being-with-other-persons and of our being-in-the-world. When the I is lost or weakened during an acute psychotic state, the “thou” might become even more relevant and fundamental source of support (cf. Buber, 1971). Thus, the therapist’s expression of her person through her stance might play a crucial role in the psychotherapy of psychosis as it could help support the recovery of a sense of self in the other (i.e., the patient).

When looking at the literature on psychotherapy of psychosis one can already derive different descriptions of a beneficial therapeutic stance for the three main strands of psychotherapy (PD, CBT and ST): PD of psychosis (cf. Lempa et al., 2017, p. 201) puts a strong emphasis on the emotional relationship between therapist and patient as a supportive framework for the reconstruction of the patient‘s ego. The therapeutic stance here seems to oscillate between closeness (e.g., openness, empathy) and distance (e.g., confronting the patient, demarcation) to the patient (Benedetti, 1983; Schlimme & Brückner, 2017). CBT for psychosis focuses essentially on developing coping skills with regards to psychotic symptoms, thereby fostering better self-management (Tarrier et al., 1997). This therapeutic stance appears to be mostly psychoeducative and instructive, but also empathic (e.g., with regards to delusional beliefs, see Mehl & Lincoln, 2014). Finally, ST of psychosis advocates a participative, collaborative and dialogical stance, in which therapist and patients relate as equals, together trying to make sense of symptoms (Smith & Karam, 2018; von Schlippe & Schweitzer, 2016).

The three main approaches of psychotherapy of psychosis advocate different therapeutic stances towards patients with psychosis. At the same time all descriptions of the therapeutic stance imply different characterizations of interpersonal therapeutic closeness and distance. Yet, a further investigation and conceptualization of helpful and unhelpful aspects characterizing the therapeutic stance in psychotherapy for psychosis is still needed.

The aim of the present study is thus to qualitatively explore psychotherapists‘ stances towards persons with psychosis. The following research questions guided our investigation:

1. How do therapists describe their own stance towards patients with psychosis, especially in terms of closeness and distance? 2. How does their stance evolve both over the professional career (also with reference to their therapeutic affiliation) and in different treatment phases? 3. How does it impact the therapeutic relationship and process (i.e., what do professionals experience as helpful/unhelpful for the therapeutic process?)?

## Method

### Participants

For this study, psychotherapists who work in the context of psychosis treatment, characterized by different academic and therapeutic backgrounds, were interviewed about their stances towards persons with psychosis during treatment. Six mental health care professionals (three women, three men) with an age range of 37 to 71 years (mean age 47) were recruited from the staff pool of three different (both out-patient and in-patient) psychiatric institutions. Participants were recruited purposely in order to represent the three main psychotherapy approaches in Germany: two therapists were trained in CBT, two in ST and two in PD. One therapist received both training in systemic therapy and psychodynamic therapy but characterized his stance and practice as being mainly based on the systemic approach and was thus included in the study representing the systemic school. Some participants also stated to have additional therapeutic training (e.g., hypnotherapy, EMDR; see Table I). Three psychotherapists held a medical degree, three a psychological one. Clinical experience in the treatment of psychosis ranged between 2,5–42 years (mean 16,9 years). The therapeutic settings covered by the psychotherapists encompassed in- and outpatient treatment, home treatment, day hospitals and an assisted living facility. Additionally, three psychotherapists also stated to have had experiences with psychosis in a non-professional context, i.e., either on their own or in their private environment (see Table I). None of the psychotherapists had received a formal diagnosis of psychosis in their lifetime.

**Table I.** Demographical data and characteristics of clinical care provided by the participants in the sample.

P^1^	Sex[F/M]	Age	Nationality	Profession	Therapeutic approach[BT^2^/ST^3^/PD^4^]	Completion of training	Further therapeutic expertise	Clinical experience with psychosis [y]	Patients receiving medication while treated	Therapeutic Setting	Experiences with psychosis in a non-professional context
1	F	40	German	Psychologist	CBT	2014	EMDR^5^ Schema therapy	8	Mostly	Outpatient, Assisted living facility	No
2	M	38	German	Physician	CBT	2021	No	7	Always	Outpatient, Home treatment	Yes^6^
3	F	37	German	Psychologist	ST	In training	No	2,5	Mostly	Inpatient, Outpatient	No
4	M	71	German	Physician	ST (main)/PD	1990	Psycho- traumatology, Hypnotherapy	42	Mixed	Inpatient	Yes^7^
5	M	59	German	Physician	PD	1997	No	32	Mostly	Inpatient, Outpatient, Day hospital, Home treatment	Yes^8^
6	F	37	German	Psychologist	PD	2018	Interdisciplinary Psychosis therapy	10	Mixed	Outpatient	No

^a^P: Participants; ^2^CBT: Cognitive behavioural therapy; ^3^ST: Systemic therapy; ^4^PD: Psychodynamic therapy; ^5^EMDR: Eye movement desensitization and reprocessing; ^6^several months persisting auditive hallucinations related to a mental stress situation; ^7^three times fever hallucinations in adolescence; ^8^among relatives or friends (personal note).

### Data collection and analysis

Participants received written information about the aim and procedure of the study. All participants provided their written informed consent and received monetary reimbursement for their participation. All data were treated anonymously and confidentially. Formal approval was obtained from the ethics committee of the Brandenburg Medical School. All steps of the study were conducted according to the national and international ethical guidelines and to the Helsinki regulations.

Before the interviews started, the participants were asked to fill out a questionnaire concerning their sociodemographic and professional backgrounds. Then semi-structured qualitative interviews were conducted (4 by EJ, 2 by ST). Interviewers were trained in and followed the quality standards of qualitative interviewing (Knox & Burkard, 2009). Interviews lasted between 54 and 72 minutes and were audio-recorded and later transcribed verbatim.

Prior to the empirical data collection, we conducted a literature research on the concept of therapeutic stance and on its application in the PD, CBT and ST of psychosis. Insights from this review were used as *sensitizing concepts* (Blumer, 1969; Charmaz, 2014) for the theoretical sampling and for developing the interview schedule. In all three therapeutic approaches, the dimension of closeness and distance seemed to play a crucial role with regards to the therapeutic stance. We thus decided to specifically ask the interviewees to describe their stance, especially on a closeness-distance continuum.

The interview schedule included open questions about three central themes: 1. The description of the therapist’s stance; 2. The formation and development of the therapists’ stance (especially with reference to their therapeutic affiliation) 3. The impact of the stance on the therapeutic relationship and process (i.e., helpful/hindering aspects). These open themes were further explored through follow-up and more detailed probing questions (see Appendix 1).

Data were analysed with the Grounded Theory method, more specifically, in the current study we adopted the constructivist approach by Charmaz (GT, Charmaz, 2014). According to a constructivist perspective, we conceive the clinical reality as being processual and co-constructed by its agents. This epistemological assumption is also coherent with the notion of stance (Kurbacher, 2017) on which the current investigation is based. The Grounded Theory Method is also coherent with our research aim, as it allows the generation of a theoretical framework stemming from the empirically generated data. The presented data analysis is part of a broader GT Process (driven by the GT principle of theoretical sampling) in which first psychotherapists and then patients were interviewed about their experience of the therapists stance. In the first step, interviews with psychotherapists were analysed until saturation of categories was reached. In the second step, interviews with patients were conducted and analysed separately. In a third step, these two perspectives were brought together into an encompassing GT model. In this paper, we present the results of the first research step including the psychotherapists perspectives.

### Data analysis

Data collection and analysis took place simultaneously. QSR-NVivo software was used to facilitate data processing. Before starting with the coding procedure, the researchers (LG and ST) listened to the interview and read the transcripts, writing down memos with the first impressions and thoughts. The analysis encompassed three steps of initial coding, focused coding and theoretical coding. Initial coding was done line-by-line with proximity to the text so that the codes were as close as possible to the participants‘ words and expressions. During this phase, researchers particularly adopted a bottom-up inductive approach: they bracketed off their assumptions by writing them down as memos and tried to be as open and as receptive as possible to the meanings arising from the text. More analytical-focused codes were then built up by continuously comparing data for similar and diverging characteristics within every single interview, grouping initial codes and choosing the most important initial codes (Charmaz, 2014, pp. 57–63). Finally, theoretical codes were built from the focused codes to interpret broader sections of the data to reach a higher analytic level of abstraction (ibid., pp. 63–66).

**Figure 1.**
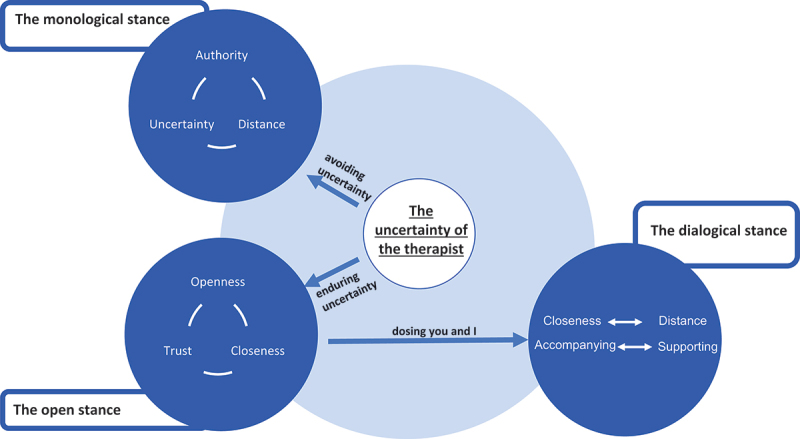
Theoretical model of therapists’ stance towards patients with psychosis.

In the second step, the theoretical codes of all interviews were clustered and pooled, in order to construct a theoretical model. Four main core categories describing the therapeutic stance were extrapolated. These categories were significantly present in all interviews and were later elaborated into a dynamic theoretical model of the therapeutic stance towards patients with psychosis. The final theoretical model was made up of 4 core categories and 5 sub-categories, describing common aspects of the stance towards patients with psychosis for all interviewees (see Figure 1). During the entire analysis, reflections, hypotheses and notes were written down in the form of memos and used to facilitate the integration of the data. The entire analysis was made in German. The final results were translated into English.

### Credibility check

LG and ST coded and analysed the interviews separately (4 LG, 2 ST) and recorded a detailed audit trail of the analysis for credibility check. Trustworthiness of the analysis was ensured through several steps. First, LG and ST respectively checked the audit trail of the interviews from the other coder and adjusted the results accordingly after thorough discussions. In the second step, two external reviewers (RE and TS) did a complete review of the audit trails. Through this review, feedback by TS as a peer researcher was especially helpful since it helped to better critically reflect upon potential blind spots of the others’ (non-user-)perspective and interpretation and thereby further increased the credibility of the results.^1^ Modifications of the final results and theoretical model were done accordingly after the review feedback.

### Analysts

Three authors of this study are, besides their research, clinical practitioners with a special interest in therapy of psychosis.

ST is a male psychiatrist and cognitive behavioural psychotherapist (38y old) with a degree in medicine (PhD) and philosophy (PhD) employed at the Department of Psychiatry and Psychotherapy, Immanuel Klinik Rüdersdorf, Germany, as a clinician and researcher. He has training in cognitive-behavioural therapy and 5 years of clinical expertise. The author has himself a biographical background of close-to psychosis experiences. This biographical background helped him to especially pay attention to statements where interviewees reflected upon their own possibility of becoming psychotic.

RE is a male psychiatry resident (31y old) who works at the department of psychiatry and psychotherapy, Immanuel Klinik Rüdersdorf, Germany, as a clinician and researcher. He started training in psychodynamic therapy 4 years ago and has 4 years of clinical expertise in psychiatry. He holds a medical doctorate in the field of neuroimaging of anxiety disorders and has recently begun to work with qualitative methods.

TS is a person with lived experience (45y old). She set off on her road to recovery many years ago and although she is not yet symptom-free, she has been working as a trainer and lecturer in the field of social psychiatry for nine years. She identified her difficulties in finding a balance between closeness and distance as triggers for psychotic episodes. During her recovery, she experienced both helpful and destructive stances from her therapists.

EJ is a female clinical psychologist (28y old) who focused on psychotherapists’ stance in the treatment of psychosis in her clinical degree. She is currently working as a clinical and psychodynamic psychologist at the department for psychiatry of the University Medicine in Halle (Saale).

LG is a female clinical psychologist and systemic psychotherapist (35y old) who works both clinically and academically at the Brandenburg Medical School. Since her doctoral degree in psychiatry she has focused her research on therapy of and on recovery from psychosis. She has also cared for a close family member, who has experienced psychosis. Since then, she has developed a conviction for the importance of interpersonal aspects in supporting recovery from psychosis and a sense of urgency for better understanding those factors. This might have led to specific attention during the analysis process for helpful and hindering aspects of the therapeutic stance.

## Results section revised

In the following section we present the four core-categories of our analysis. This analysis enabled us to distinguish three different stances towards persons with psychosis: A monological (category 2), an open (category 3) and a dialogical one (category 4). These stances are in a dynamic relation, which we ultimately illustrate with a model.

## Category 1. The uncertainty of the therapist

A basic uncertainty^2^ in the encounter with persons with psychosis pervades the participants’ descriptions of their stance. Uncertainty was partly explicitly thematized by participants and partly indirectly emerged from their narratives. It constituted a main semantic aspect in our analysis.

On the one hand, uncertainty was related to a feeling of ambivalence about ways of understanding and explaining psychosis, which immediately has relational repercussions on the stance. On the other hand, uncertainty was described as arising especially in encounters with persons in acute psychotic states. Acute psychosis triggered a sense of incomprehensibility and distance, which in turn increased insecurity.

In what follows, we describe in more detail the *feeling of uncertainty* of the psychotherapist in relation to these two aspects, which we differentiate as sub-categories.

### Feelings of ambivalence

This sub-category captures the therapists’ ambivalence in their definition or understanding of psychosis. Participants struggled in seeking a valid definition of psychosis and expressed their dissatisfaction with established diagnostic definitions.

One means a lot of things by that. One usually means that people either hallucinate or have ego disorders or are delusional (…). This is just … The concept is … clinically not properly defined. (PD1)

The lack of a shared objective definition of psychosis to rely on yielded insecurity. Consequently, the disorder was connoted as a construct to be socially negotiated in different contexts, thus being rather described as a label. Here the relational consequences of such an (conceptual) ambivalence become immediately clear. In the participants’ accounts, the definition of psychosis was described as varying according to different (relational) contexts and at the same time, as something that informs the very relational context in which therapists encounter patients. Participants for example expressed the worry for the potential detrimental effect such label can have on patients:

I am worried and I know many bad stories in which the diagnosis alone becomes part of the secondary trauma of this illness. (ST1)

Across the interviews, ambivalence also more directly concerned the etiopathogenesis of psychosis. Participants gave both biological and biographical explanations in a way that partly appeared contradictory or paradoxical. We observed in the narratives that this ambivalence lead to a sort of splitting between different views of psychosis, or even between two different “classes” of psychotic patients:

It is a whole different story whether I have someone who has something like a, say, badly progressing (…) schizophrenic disease that gets worse every year and on the other hand what Bleuler describes. Thank God there are not many cases – but there is something like “dementia praecox” – people who slip really very early THROUGH psychosis into a cognitive deficit. That is something different than when I talk to a person who has a psychotic crisis experience due to a stressful situation. (PD1)

Participants fluctuated between a deficit-oriented view of psychosis as an endogenous biological disorder of the brain, i.e., a deficit or illness emerging from “within” and from the body, and a view of it as a meaningful reaction to biographical problems and conflicts, i.e., a problem-solving attempt of the human psyche to its surrounding world. This dualistic view is mirrored by different approaches (and possibly stances) to treatment: i.e., assuming a chronicity of an illness to be treated neurobiologically and with coercive measures if necessary or making sense of psychosis though psychotherapy.

Interestingly, a similar splitting arising from a sense of ambivalence was described in relation to the very person of the psychotherapist. They seem to struggle in the search for the right way to relate to and act with psychotic patients. As it emerges in the following quote, such a struggle is enacted both in the social sphere—struggle among colleagues—and in the intrapersonal sphere—a sense of inner disintegration or uncertainty:

There were these clinics that strapped people down and there were these super clinics that were psychoanalytical and said, ‘but we are the RIGHT ones and we understand it all better and we would NEVER physically restrain a patient’. That’s all vile, all hypocritical. (laughs) So these kinds of divisions still exist. And just as you can’t divide patients into different parts - you can’t divide a therapeutic personality into different parts. (PD1)

### Acute psychosis yields uncertainty

Uncertainty in the stance of psychotherapists especially emerged in descriptions of encounters with patients in acute psychotic states.

I can only personally say for myself that people with acute psychosis always unsettle me, especially when it comes up on night duty. (CBT1)

Therapists reported difficulties dealing with unexpected and incomprehensible behaviour and with patients“ strong emotions. Acute psychosis challenged the therapists” therapeutic stance and at the same time they contributed to the formation of a different and specific stance towards patients with psychosis (see also category 4):

When the person in front of me is somehow blocked and somehow cannot answer my questions or … when facial expressions and gestures are somehow totally … frozen and plain - it is very difficult for me to establish a conversation with this person. So, these contacts on night duty in any case - so I definitely noticed that sometimes it is not easy for me to work together WITH the patients in acute situations and that this leads to insecurity, which in turn is in my view completely normal. But I have learned for myself that perhaps this (…) has contributed to my special attitude towards psychosis patients. (CBT1)

The feeling of uncertainty is mostly related to the sense of incomprehensibility and unreachability experienced in the encounter with persons in acute psychotic states. Participants experienced this very sense of distance or radical alterity as being inherent to psychosis:

By psychosis … I mean when a person perceives another reality or a reality that is significantly distant or different from the … socially shared reality. (CBT1)

When it’s really acute, I notice that I can’t really reach him. (CBT2)

Not being able to understand what is going on in psychosis also meant not being able to predict and to act accordingly, which yielded not only uncertainty but also feelings of anxiety:

Well, I can remember, back then during the time, during the internship, those were the first experiences on the acute ward, on the closed ward, where one also had to deal a lot with the acute psychotic patients. I was just still very naive. My attitude at the time was rather anxious (…) because I didn’t know exactly what was going to happen. Would they be aggressive? (…) I still didn’t understand many things, how it all works and above all [I felt] a bit of helplessness: “Yeah, what can I even do as a psychotherapist?” (CBT2)

As a consequence, psychotherapists also described distance as their reaction, as a way to protect themselves:

When it is necessary to distance myself, then I distance myself or when I … a boundary - Well, it has also something to do with boundaries. To have protection for myself and I think – I’m not really good at that. (CBT1)

Uncertainty thus also seems to arise from the experience of unreachability and incomprehensibility (and thus unpredictability) of the other person, which were felt especially in situations of acute psychosis. This feeling of uncertainty was not described as a stance per se, but as a way of being and feeling when encountering psychosis. Yet importantly the way psychotherapists dealt with their uncertainty informed two different stances, which we carved out as playing a central role in the relationship with persons with psychosis. In our analysis, we thus grouped different strategies to deal with uncertainty and the underlying relational stances described by participants in terms of avoiding or enduring uncertainty. These are described in the following two categories.

## Category 2. Avoiding uncertainty: a monological stance of “either you or I”

The interviewees described a tendency to avoid uncertainty by either relying on their theoretical knowledge and associated therapeutic interventions or by withdrawing to their given authority of their professional role. In the following, we will describe this as a monological stance. Instead of engaging in the relationship and focusing on the individual patient´s needs the therapists adopted a distanced and detached position that stressed their own characteristics and actions (e.g., the professional role and its authority, knowledge, therapeutic interventions). This focus was described as a remedy in moments of uncertainty with some participants explicitly describing it as a way to protect themselves from (and thus avoid) feelings of uncertainty and anxiety, especially at the beginning of their clinical practice (sub-category 2.1).

My stance has changed there, too. Especially at the beginning in the acute wards I went through the whole program of psychoeducation and I had to explain everything [to the patients]; paint it all so nicely on the flip chart … And then I noticed that they were not so interested – or often not so interested, but that it was more about actually wanting to be understood. (CBT2)

As it emerges in this quote, exclusively relying on one’s own theory and knowledge means to disengage from the interaction. In the example, the therapist was so concentrated on her psychoeducation that she didn’t perceive the patients’ reactions in the beginning. This example shows how a focus on themselves, on their own knowledge and abilities, helps to decrease uncertainty in the moment. Yet, the participants were critical of this stance as it increases the feeling of distance and hinders the therapeutic relationship.

The monological stance was described as rather one-sided, prescriptive and static. When assuming this stance, participants relied on knowledge rather than on their previous clinical experience. Although there were only few explicit passages in the narratives of psychotherapists describing themselves as authoritative and prescriptive, we could ground this category also on semantic and syntactic aspects that emerged in the texts. For instance, we found formulations from which transpires a connotation of the therapeutic relationship as a one-sided process, i.e., as being steered by the therapist, rather than being co-constructed. One participant for example, instead of speaking of trust as a reciprocal endeavour, used the wording “establishing trustworthy access to the patient”: an expression that suggests a one-sided intervention.

In this stance we recognize an either-or structure: either I feel overwhelmed by uncertainty by focusing on an (unpredictable, incomprehensible, unreachable) other or I re-gain my capability to act by focusing on and relying on myself (my knowledge, my authority, my role). It thus seems a monological stance in the sense of an “either you or I”, i.e., an (exclusive) reliance on the “I” to escape an unsettling “you”.

Since this detached and monological stance was described as a reaction to feelings of uncertainty, the more participants felt overwhelmed and insecure, the more they tended to assume such a stance. In these regards, we found an important difference between psychologists and physicians. In their narratives, psychologists integrated more characteristics of the respective therapeutic schools. Contrarily, in the interviews of the three physicians, a common narrative prevailed, namely a more accentuated version of the above-mentioned ambivalence and uncertainty, which was here formulated as a dilemma between respect for the patients’ autonomy and prevention of harm. This much stronger ambivalence experienced by physicians was related to the responsibility and pressure to take action due to their legal accountability in case of self-harm or harm to others. Consequently, physicians tend to rely on authority and knowledge in order to reduce uncertainty, especially in such high-risk situations. One psychiatrist described this ambivalent feeling with regards to coercive treatments, which he deems in case of a potentially harmful situation as a necessary, but highly undesired action:

I see myself in a bitter duty sometimes to – from the attitude – not being able to push myself into such liminal zones then. (…) Hopefully I don’t have to do burdensome things to prevent something worse. (ST1)

Indeed, the physicians explained that they often found it hard to weigh out the respect for a person‘s right to free decision-making in acute psychotic states and the obligation to ensure their safety and the safety of others. Here, structural and legal aspects skew the responsibility for treatment towards (if not entirely to) the therapist, thus per se creating a strong relational imbalance that might foster an authoritarian and monological stance. For example, in the following quote, this participant talks about a patient who committed suicide after discharge:

But of course, I thought afterwards, why didn’t I just restrain him and why didn’t I give him maximum sedation with medication? I noticed how badly he was doing, yes, and that means that in such situations, acute situations, you certainly look at other things, namely first of all that people are safe. (PD1)

## Category 3. Enduring uncertainty: an open stance towards the other

As an alternative way to deal with uncertainty, participants described the possibility to endure it. Enduring uncertainty meant in the first place a not-knowing stance, in which therapists accepted not finding answers or solutions within themselves and relied more on the here-and-now interaction with the patients.

But I don’t have to have the solution, and I don’t always need to have an idea right away. Instead, I can also tolerate the fact that at the moment I have no idea. As long as I talk to the patient about it. So I become more serene. (ST2)

Concretely this meant to focus on and being open to the other and to the contingencies of the interaction. Such openness was emphasized in all interviews as a core helpful aspect of the therapeutic stance towards psychosis.

And for psychoses, I think, you need a very open psychotherapeutic attitude that doesn’t focus too much on the setting, but more on spontaneity and openness. (PD1)

Being open meant for the participants being ready to take the other person seriously, to listen, accept and adapt to the person and to her idiosyncratic needs instead of following theories or guidelines. The focus is thus here mainly on the other and not on the self: I acknowledge and accept that I do not know and I don’t have a solution so I first follow you and adapt to you.

So we already spoke about the openness to what is happening and getting out of the comfort zone. That also includes (…) being able to bear the fact that you don’t have an idea right away. So when he comes up with something, I don’t immediately know: ‘Oh, we’ll use this method now‘. – But instead to bear the fact that you are speechless at first (…). I don’t have to know everything. (ST2)

Importantly, openness also meant flexibility, thinking out of the box, finding alternative ways to be in contact, such as going for a walk or doing something together instead of talking.

If someone is afraid of a psychotic threat, I don’t know, he just feels persecuted or hears … or feels irradiated at that moment and is completely panicked, right? – Then I can’t help him to calm down and think clearly when I talk to him for fifty minutes. That simply doesn’t help. Instead, I might have to give him a glass of water and say, “Now calm down and you know what, let’s go outside and look at a tree and then we’ll hold the tree together and see if that makes you less afraid”, right? (PD1)

Openness requires enduring uncertainty. Such reliance on the other person and on the contingencies of the here-and-now-situation means to be able to bear (potentially ever changing) unpredictable situations for the time being. In the views of some participants, too much openness at times related to feeling overwhelmed and even to fears. Participants thus described an inhibiting and hindering effect of giving in to uncertainty, which translated into being overwhelmed by it and feeling thereby incapacitated to offer a supportive therapeutic framework.

I think it is a pity that in principle these fears and anxieties perhaps also make it difficult for me to create a framework that the patient can, first of all, accept well. (PD2)

Yet, if bearable, enduring uncertainty allowed adapting to the other and being flexible in the moment-to-moment encounter without pre-planning interventions. It was described as a stance not based on knowledge but mainly on (clinical) experience, which is developed through time.

And you develop that in the course of your life, that you also dare and try things out and so on. Yes, so you just have to be open to what the patient brings to you, yes, but also open to your own role as a therapist. (PD1)

Importantly, an open stance was also seen as being co-constructed (in time), as something intrinsically relational, something that is shared.

And ultimately it is a question of time. So, with an individual person it is a question of how long you have known each other and then, I think, the stance becomes more flexible and, above all, it becomes shared, co-constructed between the patient and the therapist. It is no longer the performance of the therapist, so to speak. (PD2)

Openness was described as what allows and at the same time is strengthened through interpersonal contact, something that—like closeness—is co-constructed in time, in the relationship. It has to do with “being there” with “being in relation” with the other, through time. In their narrative accounts, participants thus clearly associated this stance of openness to interpersonal closeness.

Of course, I am emotionally closer to a patient who I really see weekly for years than to someone who I see maybe once every half a year and of course, I am also closer to patients with whom I—with whom I know the living situation, with whom I have perhaps already gone through ups and downs or which then we have gone through together and where the patient simply has a lot of trust in me. Exactly, and when you go through something like that, it brings you closer, especially when you also go through crises like that. (CBT2)

Being close to patients was connoted by all participants as positive and helpful for the therapeutic relationship. It was described as a necessary condition for the emergence of *trust* in the relationship. Participants emphasized the importance of trust. They described it as a co-constructed basis that on the one strengthens the relationship and on the other hand makes patients feel safe.

Well, closeness is particularly helpful in psychosis because there is trust. So especially if you have paranoid schizophrenia or people who hear voices and so on and are afraid to express that, closeness is particularly important because only then do the patients have trust (…) and they talk or come to me when they notice: “my voices are starting again at night”. This is closeness, something basic that one needs for therapy, so that they also talk to us about it … (CBT2)

Even if not explicitly stated, from the way participants talked about enduring uncertainty, we carved out a tendency to seek safety within the relationship (see quote at page XX: “I do not know but if I can talk to the patient, I feel serene.”). Thus, not only for patients but also for therapists, trust might be seen as something that gives safety and in turn might help enduring uncertainty.

## Category 4. Dialogical stance: dosing you and I

This last category is about the therapeutic stance, which participants experienced as helpful when relating to persons with psychosis. In our analysis, we grouped and clustered all descriptions of a helpful therapeutic stance and conceptualized their common core as *dialogical*. This particular stance stems from and includes the stance of openness described above (which was also described as helpful) yet it is not limited to it. The focus of an open stance is on the other person, which is what allows closeness and trust. Yet, what was in common to the descriptions of a helpful stance was indeed not a sole focus on the patient but rather a balancing act, that allows the co-presence—i.e., a dynamic *dosing* - of you and I. We found the expression “dosing you and I” from Scholz (2023) particularly fitting to describe this stance, as it points to the possibility of a co-presence of you and I, yet not as something static and absolute but as a process and ever changing dynamic. Indeed, the term “dosing“suggests different possible degrees of presence or space for the self and the other in relation. A dialogical stance is thus not only about a co-presence but also about a co-regulation of such interpersonal space. In other words, central to such a dialogical stance is the question of how much “you” and how much “I” takes interpersonal space at any given moment of a relationship.

Within this category, we differentiated three sub-categories. In the following two sub-categories, we describe more concretely what this balancing act or “dosing” means, when talking of a dialogical therapeutic stance. In the last sub-category, we differentiate an important aspect emerging from participants’ narratives that directly related to category 1: whereas in category 1 we highlighted that the stance is most challenged when encountering persons in the acute phase of psychosis, here we emphasize that a dialogical stance is most needed and most relevant exactly in these acute phases.

### Balancing closeness and distance

Although, as we mentioned in category 3, openness, and thus closeness, emerged as core helpful aspects in the psychotherapists’ view, participants also emphasized the need and helpfulness of a more distanced stance at times. Participants highlighted the importance of a dynamic movement between coming close and taking distance in the relationship with patients. They described it as a balancing act:

This is perhaps also walking the tightrope, to be very empathic and dedicated and yet to be distant. (PD2)

Some participants particularly emphasized the crucial importance of setting and negotiating interpersonal boundaries, thus focussing more on differences than on empathy:

“So that’s more in acute cases or when there is really paranoia about the whole thing, then I’m also very clear. (…) And then I still try to stay in touch and to say ‘Hey, that’s ok, your perception, that’s your illness‘, yes, right? So that’s really … like on a seesaw, back and forth. (CBT2)

The importance of distance and boundaries per se does not have the same weight as the importance of closeness across interviews, yet an emphasis on balancing between closeness and distance (in a need-adapted way) emerged as a core common topic. In category 2 we have outlined that a distanced stance is mainly characterized by a focus on oneself (at the risk of losing contact with the other) and in category 3 that a stance of closeness is characterized by a focus on the other (at the risk of losing oneself/being overwhelmed). Yet, what participants experienced as helpful, was a dialogical stance that balances between—or”doses“- the two: a dual or alternating focus on oneself and on the other, which translates in an interpersonal movement of getting closer and distancing. Consequently, it is not about being “either distant”-“or close” but about dosing both distance and closeness depending on the given contingencies and on the patients‘ needs.

Openness and closeness are thus not enough for a helpful stance. In a dialogical stance, both poles of closeness and distance acquire relevance. Yet an open stance was depicted a necessary step or entry point for enabling in the first place this dialogical oscillation: only if the therapist is open towards her patients’ experiences and needs and has gotten in touch with them can she then start to adequately and responsively shift and balance between closeness and distance.

### Balancing supporting and accompanying

A similar dialogical movement is to be found in descriptions of the therapeutic stance on a similar continuum, concerning how therapists position themselves in the relationship to patients. One side of this continuum was described as an *accompanying* stance, characterized more by listening and figuratively by “standing beside” patients.

In general, it is important for me to be a companion, insofar as we agree on a goal together. And it is also very important for me that the patients formulate the goal in such a way that I understand it well and internalize it. So that when I accompany them, I don’t somehow pursue a different goal than my patient. But that we have a goal in mind together and try to reach it together. And not that I pull them towards it or push them towards it. But that I, that they, so to speak, set the path and I am at their side. (ST2)

To “accompany” means to hold back and make space for the patient, for her goals and her ways to reach them. Instead of *doing* something, it rather means *being* with the other. In this stance, the therapist sees herself as a “companion”. She does not “push” or “pull” the patient, which suggests a rather non-interventionist stance. For the therapist this means to be ready to follow the patient, to adapt to her own way and will. Some participants here used the German word “beistehen” which literally means to stand (“stehen”) beside (“bei”). One might thus even symbolically visualize such a stance as one in which therapist and patient look in the same direction, instead of being directly the object of each other’s attention. As one participant emphasizes (ST 2), it also implies humility, giving up control and sharing responsibility.

And that gets - so that’s what I can offer the patient by being there for him, like - I don’t know - once a month, even if it’s only half an hour, and then listening and trying to somehow - to somehow find a way to deal with it with the important everyday problems that result from the mental problem. (CBT1)

On the other end of this continuum, participants described a *supporting* stance, in which the therapist is more actively engaged and (personally or professionally) contributes to the relation:

My role is (…) to support the person concerned, to listen to them and if they ask me how I can support them further, to show them the possibilities I have, and these are (…) the various psychosocial interventions offered in the outpatient clinic. (CBT1)

Although listening here also plays an important role, the focus is on what the therapist *does* (supporting, providing new perspectives or solutions). “Supporting” here means doing something for the other, in order to help, to guide and to assist her. The therapist thus brings her own input into the relationship. Yet this input is not understood as something imposed upon the other in a prescriptive way but as an offer, which the patient can still decide to accept or deny. Picking up participants’ words, in the German designation of this sub-category, we used the word “Behandler”, which means “practitioner” thus emphasizing exactly this active, practicing, and “treating” aspect of the therapist’s role. Interestingly, when talking about their support, participants emphasized professional rather than personal contributions to the relationship.

Importantly, the relation and continuous balancing between these two aspects—supporting and accompanying—were emphasized as a crucial aspect of a helpful therapeutic stance.

More like a companion … like a companion and – and supporter, less as an agent of change or healer. (CBT2)

Here, we can recognize a dialogical structure as well: instead of shifting between either an prescriptive “I” or an unsettling “you”, a balance between accompanying and supporting enables a space for *both* you and I. When accompanying, the therapist makes spaces for the patient, yet she does not only follow (or withdraw from the interaction): she is present and “stands beside”. By supporting, the therapist takes up more space in the interaction by showing options, yet in a non-prescriptive way that respects and adapts to the patient’s will and needs. In our analysis, we carved out this dialogical co-presence of you and I in the therapeutic stance as the core of what was experienced as helpful.

That it is totally important to LISTEN to these people, who first need … more time to build up trust. And maybe they still need someone to support them in their decision-making and maybe show them the options again. (ST2)

Again, a dialogical stance is not conceived as a static co-presence: it is a dynamic movement. In this subtheme, this movement is depicted in the expression “dosing you and I”: a continuous adjustment between being with and making space for the other and taking up more space in order to offer support.

### Acute psychosis needs dialogical stance

Although challenged, the stance in itself was considered as a particularly important therapeutic aspect in acute phases. Therapists reported that pre-planned interventions are not very helpful in the acute phases. Instead, they stressed that in these moments the main therapeutic interventions are yielded through the very therapeutic stance (sub-category 3.2). The dialogical stance—with its twofold movement between closeness and distance, accompanying and supporting—was especially experienced as crucial and helpful in relating to persons in acute psychotic states.

But I also find that when the person gets into a crisis, and I have this open stance and the accepting stance, then something changes in the stance insofar as I then also, very concretely – so when it really gets into an acute crisis with psychotic patients, that I then also very concretely, draw boundaries – such boundaries as “this is your perception of things now, I have a different perception.” (CBT2)

Indeed, when describing a helpful stance that is specific to psychosis, participants talked particularly of acute phases (see e.g., quote at p. 11). This subtheme mirrors and complements sub-theme 1.3.: in both cases participants emphasized an important difference in the stance when encountering persons in acute or non-acute psychotic phases. Acute psychosis seems to be the moment in which the stance is most challenged (sub-category 1.3) and at the same time the moment in which the stance—i.e., a dialogical stance—is most needed.

## A theoretical model of the therapeutic stance towards persons with psychosis

Although the four categories present distinct aspects of participants’ experience and descriptions of different stances, they are tightly related to each other. In a final analytic step, we explored and unfolded these dynamic relations and developed a theoretical model on psychotherapists’ stance towards persons with psychosis, which we present in Figure 1.

We conceived the first category “the uncertainty of the therapist” (core category 1, at the centre of the model) as one of the main sources of therapists’ stance. When encountering psychosis (i.e., persons with psychosis but even the very concept or idea of psychosis) psychotherapists experience strong feelings of uncertainty, which we have identified as partly arising from a sense of ambivalence (sub-category 1.1.). Indeed, the difficulty of the therapists to develop a shared and coherent conceptual framework for the phenomenon of psychosis may cause uncertainty about what to do in the treatment and how to relate to the individual patient. We identified another possible source (or amplifier) for such uncertainty in the acute phases of psychosis, which were experienced as particularly challenging (sub-category 1.2.). Participants associated a sense of distance, unreachability, incomprehensibility and thus also unpredictability to the very psychotic condition—and even more so when the psychotic condition is acute.

Even if uncertainty is not (yet) a stance per se, it pervaded the experience of psychotherapists towards psychosis and informed the formation of specific stances. The therapists’ inner struggle and uncertainty is thus the starting point of our model.^3^ Depending on how therapists deal with their uncertainty, they can assume two different stances, which we depict in category 2 and 3 (at the left hand side of the model): a monological stance when avoiding uncertainty and an open stance when enduring it.

Avoiding uncertainty by seeking security in theoretical explanations, pre-planned therapy plans, one’s professional role or authority was summarized within the umbrella term of a monological stance. Indeed, here the use of professional knowledge or interventions is implemented one-sidedly, without adapting to the patients’ needs and reactions. By assuming this stance the therapist focuses mainly on herself, her knowledge, her professional role, her decisions. Although this might at first have a reassuring effect, in our theoretical model we highlight how such a stance can lead to a vicious circle that—on the long term—increases the very uncertainty it was set to avoid. By reacting authoritatively and narrowing her focus on theories and guidelines, the therapist distances herself from the patient. The distancing of the psychotherapist might be mirrored at the same time by a distancing and deeper isolation of the patient, who at this point might not feel seen or taken seriously and potentially retreats in turn even further in her (for the therapist incomprehensible and unsettling) psychosis. We have already highlighted in category 1.2. (“acute psychosis yields uncertainty”) how the experience of interpersonal distance, and thus of unreachability and incomprehensibility of the other, yields and increases insecurity. The mutual distancing of therapist and patient, triggered by a monological stance, can thus create even more insecurity and uncertainty, which eventually reinforces the therapist’s tendency to act prescriptively in order to feel more secure. This self-sustaining vicious circle might thus result in an increasing interpersonal gap between therapist and patient, and a deepening of uncertainty at the interpersonal level. From here, each of them might increasingly seek their safety in isolation: the therapist in her theories and standard interventions and the patient in her silence and non-disclosure about her psychotic experiences (or even in the very delusions).

The alternative reaction to the felt uncertainty is to endure it instead of avoiding it. When the therapist manages to endure and tolerate uncertainty a virtuous circle might evolve. We have highlighted in category 3 that enduring uncertainty means being open to the other person and to the here-and-now-contingencies of the interaction. Instead of seeking security in her own knowledge, the therapist assumes a not-knowing stance and focuses mainly on the patient. Such a stance of openness allows being in contact with the patient (through listening, adapting, following, acknowledging etc.), which in turn leads—through time and increased sharing of experience—to an increased feeling of closeness. Importantly, closeness strengthens the relationship and enables trust. Here too, we see a looping effect, since the co-construction of a trusting relation (as something that gives stability and security) might exactly be what in turn allows the therapist to endure uncertainty more easily and thus to be more open and to focus more on the other person. The virtuous circle thus starts from uncertainty, goes through openness to closeness and ends in trust feeding back into reducing uncertainty and thus making the circle at every round smoother and easier. Here, it is important to notice that this virtuous circle does not rely on theoretical expertise, but is mainly based on time, growing experience and most importantly fostered by the co-construction in the relationship.

On the right hand side of our model, we depict what we define as a helpful stance towards persons with psychosis: the dialogical stance. Openness and closeness per se are not enough but are conceived as a necessary basis for enabling a dialogical stance. Indeed, even if a dialogical stance entails both poles of closeness and distance, to first distance oneself would go with the risk of disconnection (see vicious circle in category 2). Openness, on the other hand, is characterized mainly by a focus on the patient, which not only increases closeness but is also crucial to be able to dialogically balance between closeness and distance in a need-adapted and patient-centred way.

In contrast to the “either-or” structure or a monological and authoritarian stance, we characterized the dialogical stance as a “both-and” of self and other, and more specifically, as dynamical “dosing” of you and I.

In our analysis, we characterize the dialogical stance more specifically as a “dosing” of interpersonal closeness and distance. It implies an oscillation between getting close and setting boundaries, or better, the very possibility of balancing both aspects at the same time: e.g., allowing closeness despite the necessary boundaries or setting boundaries within the context of closeness—and this at the same time always in an open connection with the experience and needs of the patient. Similarly, the dialogical stance is characterized as “dosing” of two stances: accompanying and supporting patients. By “accompanying”, the therapists step aside and make space for the patient, yet still being there for her. By “supporting”, she acts more proactively and brings her own contribution to the relationship (e.g., by making therapeutic offers), yet without prescribing or authoritatively imposing it on the patient. In both descriptions of the dialogical stance, we can recognize a co-presence of you and I to different degrees, thus, a dialogical structure.

On the whole, our model describes the dynamical transformation from insecurity and ambivalence, which gives rise to a monological stance, into a dialogical, more balanced stance. More concretely, the monological stance is based on an “either-or-structure”: either I rely on my authority or I am unsettled by your unpredictability. These mutually exclusive poles are transformed and integrated into the “both-end” structure of the dialogical stance: I can be close without losing myself and distant without losing contact—I can make space for you and still be present and I can take space in the relationship without imposing myself. This transformation is enabled by a stance of openness that in the first place allows the “dosing” of self and other in dialogue in a need-adapted and responsive way.

## Discussion

The aim of this study was to investigate psychotherapists’ stance towards patients with psychosis on a continuum between closeness and distance; more specifically we also aimed to find out differences in the stance related to the therapeutic orientations and the phases of therapy. Finally, the therapeutic stance was explored with regards to hindering and helpful factors. Drawing on the current state of the literature, we especially focused, at the onset of our study, on the psychotherapist’s psychodynamic, cognitive-behavioural or systemic orientation and the possible respective differences in the stance. We did so since, as we explained in the introduction, one can derive different definitions of a beneficial therapeutic stance for these three main strands of psychotherapy. Yet, interestingly, our results show that therapeutic orientation presumably only has little impact on the stance towards persons with psychosis: despite different therapeutic techniques and concepts of the three psychotherapy approaches, we found more common ground than differences regarding the therapeutic stance in daily interactions.

Our results show that the therapists‘ stance was characterized essentially by *insecurity and uncertainty*. A first helpful stance was based on tolerating uncertainty, which allowed a movement from uncertainty to trust through a stance of openness. Interpersonal closeness played a central role in this movement. Interestingly, whereas being open to the experience of the other was repeatedly mentioned as an important characteristic of the therapist’s stance, fostering closeness, participants did not speak of opening up themselves as a person to patients in the process of getting close. The meaning of this apparent imbalance for the therapeutic process needs to be further investigated.

Based upon and at the same time going beyond openness, a core helpful stance was described in terms of a dialogical stance, that allows a co-presence or co-regulation of self and other in the interaction. The expression “dosing you and I“, used to describe such a dialogical stance, has been introduced by Scholz (2023) to describe her own experience of interpersonal relations during psychosis. At the same time, based on our results, it also well depicts the oscillation or movement entailed in the dialogical stance of the therapist. The dialogical stance thus seems to tackle exactly an interpersonal dynamic that seems to be crucial in psychotic experience. Scholz (2023) describes “dosing you and I” - which she also refers to as a balance between connection to and separation from others—as what supports the stability and coherence of the self and enables social interactions. Psychotic experience is thus characterized by a (temporary) incapability or impossibility of dosing self and other, when being in relation. A dialogical stance might thus offer a sort of social scaffold or support that helps the person keep “dosing you and I”, i.e., balance between connection and separation, when she cannot do it on her own. This is coherent with several theories of schizophrenia that conceive it as a disturbance of the self and intersubjectivity, especially in phenomenological and psychodynamic traditions (Lempa et al., 2017; Schlimme & Brückner, 2017; Thoma et al., 2021; Van Duppen, 2017). The importance of this interpersonal and dialogical aspect also confirms current research outcomes in therapy for psychosis, where the therapeutic relationship—even more than for other patient groups—emerges as playing a key therapeutic role (Frank & Gunderson, 1990; Goldsmith et al., 2015; Shattock et al., 2018). More specifically, da Costa et al. (2020) and Shattock et al. (2018) highlight the role of genuineness, flexibility, availability, as well as empathy on the therapist’s side. Our model confirms these findings, whereby availability and empathy might be understood as forms of receptive and emotional openness.

Yet in our study, we also show that aspects such as empathy and availability, that can be generally subsumed under a stance of openness, are not enough. A helpful stance was indeed described as dialogical, i.e., thus „dosing“both openness and closedness. Moreover, our outcomes allow to expand and deepen our understanding of these previous studies in that they offer a description of how such a dialogical stance can concretely be achieved in clinical practice: It means to constantly balance between both actively supporting patients by offering and showing possibilities (without being prescriptive or authoritarian) and “standing beside them”, holding back and being receptive (without however losing agency).

Even if the word “dialogical” was not used by participants, we believe that it fittingly describes the helpful stance at the core of our results. This is also coherent with literature characterizing the core of the therapeutic work in the therapy of psychosis as a dialogical process (Lysaker et al., 2001, Galbusera & Kyselo, 2019).

Our model also emphasizes the relevance of *time* and experience for a helpful therapeutic stance (and thus a good therapeutic alliance) in terms of repeatedly sharing experiences with patients. Even though this might seem obvious, we found that developing a therapeutic stance that benefits and fosters a good therapeutic relationship is neither something inherent from the start nor can it be learned from textbooks. Only through repeated experience can therapists learn to endure ambivalence and to be open and flexible towards the patients’ suffering and needs, especially in the case of psychosis (cf. Westermann et al., 2015). This finding corroborates results from studies on the role of time for the development of beneficial therapeutic relationships (Freedman et al., 1999; Parish & Eagle, 2003).

Interestingly, in terms of differences between attitudes, we found that the professional background in medicine or psychology has more influence on the therapeutic stance than the therapeutic affiliation. Indeed, ambivalence and uncertainty emerged much more prominently in the experience of psychiatrists, arguably because of the different exposures to acute clinical situations and to the different responsibilities in those situations. In fact, psychiatrists were much more concerned with moral questions related to the need and use of coercive measures. Although participants emphasized the importance of time (and thus growing therapeutic experience) for developing more tolerance of uncertainty as well as an open and flexible stance, older and experienced psychiatrists (59 and 71) still reported to regularly struggle with their own stance, especially in acute situations. The particular challenge of acute situations these psychiatrists reported may also be due to the corresponding treatment setting. Patients in acute states are mainly encountered in hospitalized and thus often highly structured contexts, while stable patients are mainly treated in outpatient settings, which allow for greater flexibility of the relationship design and the construction and adaptation of a therapeutic stance. This would partly echo Farrelly and Lester’s (2014) finding that service structures and the “greater accountability of professionals to a wider context” with corresponding “concerns about malpractice claims” may encourage a more protective and defensive relationship arrangement.

Yet, even beyond the difference between medical and psychological psychotherapists and the corresponding treatment settings, our outcomes show that encountering psychotic patients in acute or non-acute states by itself has a strong effect on the professionals’ stance. Whereas working with patients in more acute states feeds into the therapists’ ambivalence and uncertainty, patients appearing as more autonomous and stable seemed to foster a rather confident and open therapeutic stance and the capacity to endure uncertainty. At the same time, whereas in stable phases therapists could make use of more therapeutic techniques and classical “talking therapy”, it was in the acute phases that the stance per se became especially important, sometimes even the main or only possible therapeutic means. Such balancing between different therapeutic techniques and positions might be thus considered a core therapeutic skill in the treatment of psychosis, especially in the more challenging situation of acute psychotic states. Yet only little research has been conducted on this topic. Borchers et al. (2014) suggested that tolerating uncertainty in situations in which patients experience acute states might especially have beneficial effects on the course of therapy if this uncertainty is addressed properly within the therapeutic relationship. This however requires proper training and a contextual framework in which the health care professionals are able to discuss and reflect on their ambivalent experiences and their therapeutic stance openly (Hamm et al., 2016).

## Limitations and perspectives for future research

One limitation of this study is that it, despite being user-controlled, only includes professionals’ experiences and definitions of the therapeutic stance. As mentioned above, psychotherapists’ statements were diverse yet often depicted the stance as something personal. One might critically argue that phenomena such as openness, closeness and trust are always reciprocal and interpersonal. One risk of these results—based on the participants’ narratives—might thus be to depict a concept of stance that is yet too individualistic. One might also wonder to what extent an authentic expression of the therapist, in the sense of becoming perceptible and tangible as a person with her vulnerability, might be a necessary component for such reciprocal relation (von Peter & Schulz, 2018; see also Galbusera et al., 2021). Further, our analysis did not include the perspective of other professionals (nurses, social workers etc.) and of family members. As we have seen, the stance towards psychosis is strongly influenced by the way in which someone is exposed to acute situations with persons with psychosis and to the degree of responsibility one is given in a certain setting. All these factors (acuteness, responsibility and setting) vary depending on the role one has as a professional or family member/relative. Therefore, the influence of different roles and of settings should be further investigated. For this question, innovative forms of treatment such as the *Open Dialogue approach* or home treatment are of special interest since they involve a change in setting, a greater sharing of responsibility and aim at reducing coercive measures predominantly in acute phases of psychosis (Seikkula & Olson, 2003). The analysis presented in this paper was the first step of a Grounded Theory study, which, by using theoretical sampling, will explore these different roles and perspectives. This report is thus to be considered a crucial and yet incomplete (first) part of this research endeavour.

Another point is the specificity of the professional stance: The professionals we interviewed claimed their stance to be both specific to psychosis but rooted in a more general therapeutic stance not depending on a specific disorder or diagnosis. However, we did not follow up on this connection and dynamic between a stance specific to psychosis and a more general stance. This is why further research is needed to validate and describe these two forms of stance and to understand what might lead to a change of stance with regards to other mental disorders such as, e.g., depression or anxiety disorders. This then points to the need to more precisely define the therapeutic stance in its different facets, i.e., in its more general (as e.g., “what actually makes a stance *therapeutic*?”) and disorder-related aspects. Finally, this could help to implement and teach appropriate and helpful therapeutic stances in psychotherapy training.

Despite the aforementioned need for further research, our preliminary findings could already be seen as an incentive to focus on practical experiences in psychotherapy training when it comes to acquiring a therapeutic stance. Since this stance is not necessarily bound to specific therapeutic schools, teaching and the examination therapeutic stances could be an important juncture and impulse for exchange between different psychotherapeutic approaches and thus to overcome traditional divisions between schools and concepts.

## Notes

1. This was especially the case with regards to interviewees‘ statements on personal openness.

2. The interviewees used the word “Unsicherheit“which in German can refer to both uncertainty in terms of not being sure of something (e.g., oneself, i.e., as “Selbstunsicherheit“) and insecurity (the German “Sicherheit“meaning security). Despite this semantic ambivalence the interviewees for the very most part implied the first meaning when speaking of “Unsicherheit“which is why we translated it with uncertainty. Still it should be noted that the latter dimension of feeling insecure was always tacitly present to us when the interviewees used the word in German, especially when speaking of coercive measures and closed wards.

3. This uncertainty and inner struggle is not only a source but also an integrative part of the stance. Indeed, patients can immediately perceive it in the relationship with therapists. Interestingly, in one focus group with persons with psychosis, the first answer to the question”how do you experience your therapist?“was:”they are overwhelmed“(This data was collected within the same study but it is not published yet).

## Appendix 1

### Interview Schedule

**Table:**

1	From a general point of view, what do you understand by psychosis?
2	How would you describe and characterize your personal therapeutic stance towards people with psychosis?
3	How has your stance towards people with psychosis evolved?
4	What do you understand by closeness and distance in the therapeutic setting?
5	Would you say that your stance is consistent with that of your therapeutic school?
6	Is your stance towards people with psychosis disorder-specific?
7	Can you describe if and how the stance changes in the therapeutic process?
8	Are there moments in psychotherapy when you have to explicitly rely on your stance?
9	In your experience, are there certain stances that are helpful or hindering?
